# Tracking the Deposition and Sources of Soil Carbon and Nitrogen in Highly Eroded Hilly-Gully Watershed in Northeastern China

**DOI:** 10.3390/ijerph18062971

**Published:** 2021-03-14

**Authors:** Na Li, Yanqing Zhang, Zhanxiang Sun, John Yang, Enke Liu, Chunqian Li, Fengming Li

**Affiliations:** 1 College of Land and Environment, Shenyang Agricultural University, Shenyang 110866, China; c514658@hotmail.com; 2 Key Laboratory of Dryland Agriculture, Ministry of Agriculture, Beijing 110120, China; liuenke@caas.cn; 3 Liaoning Dry Land Agricultural and Forestry Research Institute, Chaoyang 122000, China; lichunqian@126.com (C.L.); lifengming81@126.com (F.L.); 4 Liaoning Academy of Agricultural Sciences, Shenyang 110866, China; 5 Department of Agriculture & Environmental Sciences & Cooperative Research, Lincoln University of Missouri, Jefferson City, MO 65102, USA; yangj@lincolnu.edu

**Keywords:** soil carbon, soil nitrogen, soil erosion, hilly-gully watershed, source tracking

## Abstract

Understanding the deposition and tracking the source of soil organic carbon (C) and nitrogen (N) within agricultural watersheds are critical for assessing soil C and N budgets and developing watershed-specific best management practices. Few studies have been conducted and reported on highly eroded hilly-gully watersheds. In this field study, a constructed dam-controlled hilly-gully watershed in northeastern China was selected to identify the sources of soil C and N losses. Soils at various land uses and landscape positions, and sediments near the constructed dam, were collected and analyzed for selected physiochemical properties, total organic carbon (TOC), total nitrogen (TN), and stable isotopes (^13^C and ^15^N). Soil C and N loss and deposition in the watershed were assessed and the relative contributions of each source quantified by a stable isotope mixing model (SIAR). Results indicated that soil C loss was primarily from cropland, accounting for 58.75%, followed by gully (25.49%), forest (9.2%), and grassland (6.49%). Soil N loss was similar to soil C, with cropland contribution of 80.58%, gully of 10.30%, grassland of 7.54%, and forest of 1.59%. The C and N deposition gradually decreased along the direction of the runoff pathway near the constructed dam, and the deposited C and N from cropland and gullies showed an order: middle-dam > bottom-dam > upper-dam and upper-dam > bottom-dam > middle-dam, respectively. A high correlation between soil TOC or TN and the sediment properties suggested that the deposition conditions could be the major factors affecting the C and N pools in the sedimentary zones. This study would provide a scientific insight to develop effective management practices for soil erosion and nutrient loss control in highly eroded agriculture watersheds.

## 1. Introduction

Soil erosion caused by surface water runoff and sediment transport is a major natural process resulting in land degradation and water pollution in the world. In agricultural watersheds, topsoil and nutrient losses such as carbon (C) and nitrogen (N) by soil erosion could result in reduced soil productivity and nutrient enrichment in aquatic systems, which has become a major concern for food production, sustainable agriculture, and environmental sustainability [1]. The soil erosion process is largely dependent on landscape, land use, and weather conditions. Recently, accelerated soil erosion, or soil C and N loss, was reported due to climate change and intensive agricultural operations [2,3]. With a growing population and increased food demand, developing best management strategies for soil erosion and nutrient loss control, especially in highly eroded regions, has become a challenge, and the investigation of the mechanisms of soil erosion and nutrient loss processes is urgently needed, in an effort to sustain agricultural production and protect the soil-water ecosystem.

Soil organic C and N are important elements, or nutrients, that support plant and microbial growth and maintain soil ecological functions. The loss of soil organic C and N by surface erosion would negatively impact soil health and productivity and threaten stream water quality and environmental sustainability. Soil capacity as a source or sink of C and N in agricultural watersheds was largely dependent on land use, soil erodibility, and fate of C and N [4]. It is well known that increased forest and grassland could reduce soil erosion and enhance the storage of terrestrial C and N. Despite a widespread recognition of the importance of terrestrial C sequestration, the role of soil erosion in terrestrial C release for climate change regulation still remains largely uncertain [5]. The loss of soil C and N from eroded landscapes could be also re-deposited in sediments within watersheds. However, the fate of removed C or N and the source of redeposited C or N within a complex watershed are rarely studied and largely unknown.

Soil C and N exist in several chemical fractions with various solubility, which could affect the mobility of soil C and N in ecosystems. Topsoil from sheet erosion was reported to contain relatively high C and N contents with a large fraction of active or easily decomposable organic matter (OM) [5,6]. In a watershed scale, most soil C and N fractions were able to migrate with soil particle transport, leading to the average enrichment ratio of >1.0 [7]. The erosion stability of soil OM may also vary with landscape positions within a watershed [8]; for example, alluvial soil usually contained relatively high fractions of soluble OM. Thus, in order to understand the mechanisms of soil erosion or C and N transport processes, it is critical to quantify the C and N fluxes between upland and lowland, identify the C and N source in deposited sediments, and determine the C and N budgets within highly eroded watersheds.

Soil C or N stable isotope ratio (δ^13^C, δ^15^N) and composition have been used as a powerful tool to track the C or N source and contributions to sediments or particulate organics output of a watershed [9]. This is due to the specific signature composition of C or N in source pools that could be resulted in various ecosystems by differential isotopic fractionation during the physical, chemical, and biological processes of C and N cycling [7,10]. In addition, the C/N ratio was applied as a supplemental tracer to identify the source of soil OM [11,12]. The mass-balance mixing model was reported to quantify the proportional contributions of soil C and N sources within ecosystems [13,14]. Nevertheless, this model could also introduce uncertainty if multiple sources and isotope ratios were involved during soil erosion or biogeochemical processes. Recently, the stable isotope analysis in R (SIAR) model designed within a Bayesian framework was reported to study the probability distribution of source contributions in a mixture with a clearly stated uncertainty [15,16], which has been widely applied in ecological research such as food-web analyses [17].

The western Liaoning province of northeastern China is a mountainous and hilly region, where mountains and hills account for about 70% of total agricultural lands. About 49.4% of the 2,090,600 ha hilly area, 41.6% of the region, has become a severely eroded area in the region because of the highly sloped landscape. The soil erosion and soil C and N losses in this fragile ecosystem not only seriously decreased soil productivity and quality, but also affected the biogeochemical cycles of C and N in terrestrial and aquatic ecosystems. Since the 1970s, several major water and soil conservation practices have been implemented to control soil erosion in the region: one was to convert >25° of slope lands into forest or grassland and another was to construct dams in gullies and creeks. Such conservation practices have effectively reduced soil erosion and controlled soil nutrient loss. Constructed dams have served as a sink zone not only to deposit soil particles from upstream, but also store a large amount of soil C and N.

In this study, a small, highly eroded watershed in the hilly region of western Liaoning province was selected to investigate the mechanisms of soil erosion and soil C and N deposition processes and identify the C and N sources. Specific objectives were to: (i) determine spatial variations in soil C and N at various landscape positions; (ii) quantify the impact of soil erosion on soil C and N deposition within the watershed; and (iii) identify the source and contribution of soil C and N in sediments by the SIAR mixing model.

## 2. Materials and Methods

### 2.1. Site Description

**Figure 1:**
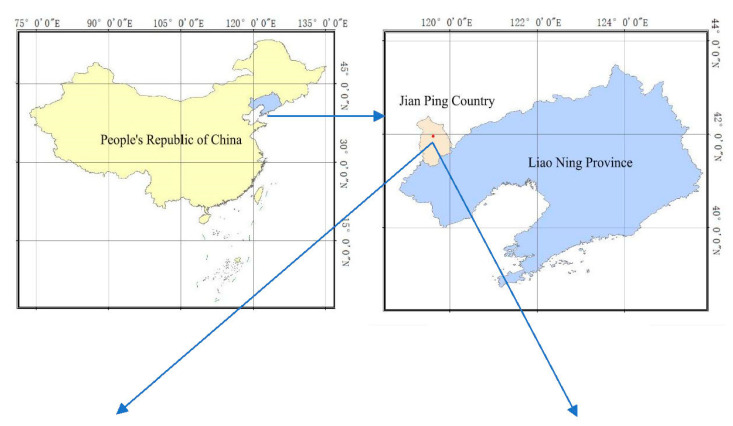

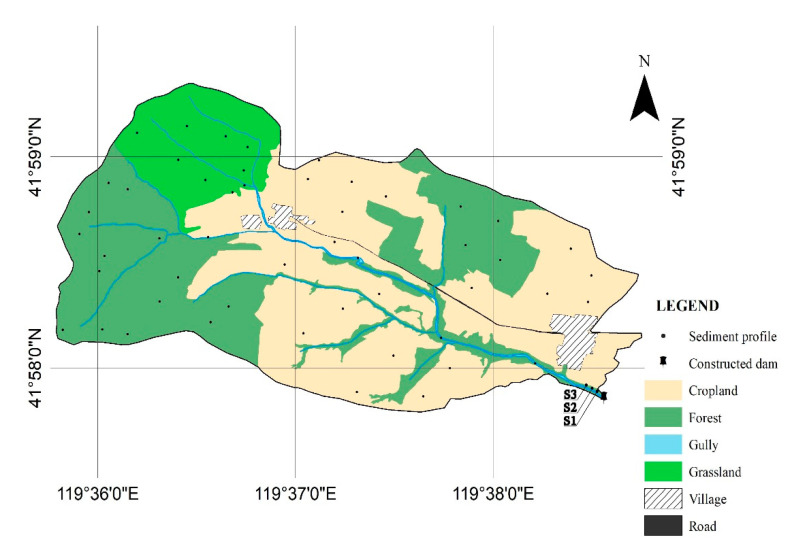
Site location, land uses, and sampling points (dots) of studied watershed.

The study site selected was a small Taipinggou watershed located in Chaoyang City, western Liaoning province, as shown in Figure 1. The area covers 17.5 km^2^ under the north temperate continental monsoon zone of arid and semi-arid climate, with annual average temperature of 8 °C, average annual rainfall of 502 mm, and annual evaporation of 2041 mm. 80% of annual rainfall is concentrated between June and September, with intensive and short-term heavy rains. The site is characterized as a hilly area with average 80% slope lands and cinnamon soil (brown podzolic soil). The land uses in this watershed include major cropland (5–10% slope) and forest (5–15% slope), and minor grassland (5–8% slope) and gully (7–10% slope) (Figure 1). Its main crop is corn under continuous farming practices.

Soil in this watershed is subject to dominant hydraulic and gravity erosion because of sloped landscapes. Since the 1970s, some steep sloped croplands were converted to forest or grasslands and a dam was constructed at the watershed outlet as soil and water conservation measures to control soil erosion and retain soil sediment [18]. Currently, there are about 1-m thick deposited soil sediments near the constructed dam.

### 2.2. Sample Collection and Preparation

Topsoil (0–10 cm) that were removable by erosion were collected with a triplication at each upper, middle and bottom position of the slope lands from each land use (cropland, forest, grassland and gully) in September 2018. At each sampling point as shown in Figure 1, three replicates of soil samples were randomly collected. A total of 165 soil samples were collected from each land use representing cropland (19 × 3), forest (14 × 3), grassland (12 × 3) and gully (10 × 3). In addition, three replicates of undisturbed 0–10 cm soil cores were also collected from the same sampling points using a standard 100-cm^3^ steel ring for soil bulk density (BD) and water content (SWC) analyses.

Sediment samples were collected from the deposited zone at the front of the constructed dam. One core, 1-m long and 7-cm diameter, was drilled by a soil sampler at each of the upper-dam (S1), middle-dam (S2), and bottom-dam (S3) positions as shown in Figure 1. The sediment cores were divided into a 10 cm increments and a total of 90 samples were collected (3 cores, 10 increments, and 3 replicates). In addition, the width, length, and thickness of the sediment wedge were measured, and the original extracted cores were used for soil BD and SWC analyses.

All sampling points were positioned using GPS Trimble GeoXM with a differential correction. The collected samples were placed into plastic bags and transported immediately to laboratory for physical and chemical analysis.

### 2.3. Laboratory Analysis

Before analysis, visual roots and stones in the collected samples were removed manually. All samples were air-dried and divided into two subsamples. One subsample was passed through a 2-mm sieve for soil texture and pH measurements, and another through a 0.15-mm sieve for total organic carbon (TOC), total nitrogen (TN) and stable isotope ^13^C and ^15^N analyses. Soil pH was measured at a soil/water ratio of 1:2.5 by a HI 3221 pH meter (Hanna Instruments Inc., USA). Soil particle size was determined at a range of 0.02 to 2000 μm by a Coulter LS200 laser particle analyzer, following organic matter removal by H_2_O_2_ and particle dispersion by hexametaphosphate. Soil BD was measured by volumetric ring method and SWC determined gravimetrically by being oven-dried at 105 °C for 48 h. Soil TOC, TN, and C/N stable isotopes were analyzed by the Environmental Stable Isotope Laboratory of Chinese Academy of Agricultural Sciences, with a Vario PYRO cube elemental analyzer attached to an IRMS (Isoprime100, Isoprime, UK). The international standards of IAEA-N1, IAEA-N3, USGS24 and USGS41 were included in the sample analysis for QA/QC, with a triplicate sample precision of <0.2‰. The average sample precision for δ^13^C and δ^15^ N was 0.01‰.

### 2.4. Statistical Analyses

T test and Mann-Whitney U test were performed to evaluate δ^13^C, δ^15^N, TOC and TN to distinguish sources, and ANOVA was used to compare means of soil properties (BD, pH, WC, clay, silt, and sand content) among land uses and landscape locations, and means of TOC, TN, δ^13^C, δ^15^N, and C/N ratio among source soils and sediments at a significance level of *p <* 0.05. All statistical tests were performed using SPSS version 18.0 (SPSS Inc., Chicago, IL, USA) and data plotted by Original Pro 2016. Principal Component analysis was performed on source soil and sediment properties (BD, pH, WC, TOC, TN, clay, silt, and sand content) by Original Pro 2016 to identify the principle factors responsible for the C and N transport in the watershed.

### 2.5. SIAR Source Appointment Mixing Model

SIAR mixing model is a statistics-based model comprised of the Bayesian isotope mixing model and Markov chain Monte Carlo (MCMC) model for determining the proportional contribution of sources to a mixture. The output of the SIAR model includes a posteriori distribution that represents the true probability density and overall residual term of mixed contribution of sources. The model has been widely employed in stable isotope environmental research to successfully track soil erosion and soil C and N transport within a watershed scale [19,20,21,22]. A detailed description of this model can be found [23].

To assess the contribution of C or N sources in various land uses, the SIAR model used δ^13^C, δ^15^N, TOC, and TN data to estimate the source contribution to the sediment in the watershed and correct the concentration dependence of δ^13^C and δ^15^N. In order to determine the relative contribution of different sources to TOC and TN in the sediments, the contribution of each source to TOC or TN output was calculated as follows [23,24]:

(1) $$\%Esource_{i}=\frac{source_{i}\times \%cont_{i}}{\sum_{i=1}^{i=4}source_{i}\times \%cont_{i}}\;\times \;100$$

where %*Esource_i_* was the percentage of contribution of each source; *i* represented the C or N source, 1 to 4 of land uses studied (cropland, forest, grassland and gully); *source_i_* was the average of TOC or TN content of each source; and %*cont_i_* was the output of SIAR model representing *source_i_* contribution to the average percentage of sediment output. In SIAR model, SGUM v0.96 was used to calculate the propagated standard deviation of TOC or TN contribution from each source [23,25], and the SIAR model operation followed the procedures described in (http://sethnewsome.org/sethnewsome/EE_files/SIAR%20for%20Ecologists.pdf (accessed on 6 December 2020)).

## 3. Results

### 3.1. Physiochemical Properties

**Figure 2:**
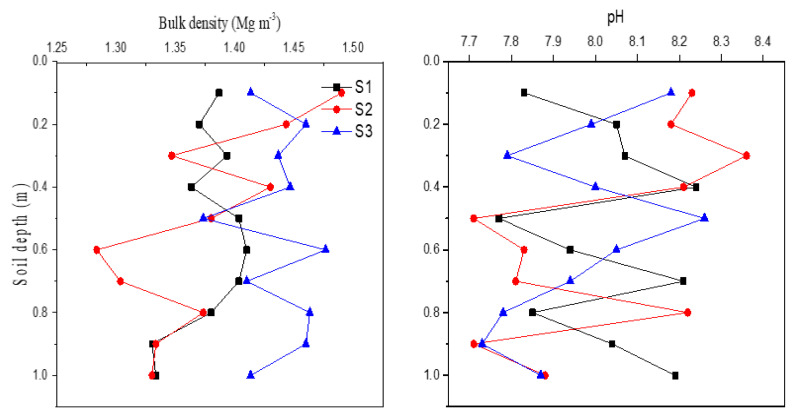
Measurement of bulk density and pH in sediment cores near the constructed dam. (S1: upper-dam; S2: middle-dam; S3: bottom-dam positions).

Selected physiochemical properties of soils in various land uses and landscape positions were presented in Table 1. Soil BD was significantly different among cropland, forest, grassland, and gully, with the highest in forest (1.38 g cm^−3^) and the lowest in gully (1.18 g cm^−3^). Even though there was no significant difference found among landscape positions, a slightly higher BD value was observed at lower landscape positions. All soils were found slightly alkaline with the highest pH in grassland soil (8.10) and the lowest in cropland soil (7.47). Soil texture was primarily silt loam in the watershed, but the particle size composition varied with land use and landscape position. Forest soil contained more sand and less clay than any other soils, and the soils at lower landscape positions had more silt and clay contents as compared with those at upper positions. Measurements of soil particle size composition were generally consistent with soil BD data among landscape positions. The relatively higher soil clay content and BD at the lower landscapes suggested that this watershed could be subjected to soil erosion moving fine soil particles from upper to lower landscape positions to some extent. Soil sediments near the constructed dam generally had relatively higher BD and pH values compared with the source soils. However, there was no clear distribution pattern observed in the profiles (Figure 2).

### 3.2. C-N Content and Stable Isotopic Characteristics

Measurements of TOC and TN in the soils and sediments presented in Table 2 indicated that TOC and TN contents significantly differed among land uses and between source soils and sediments. Forest soil contained the highest TOC, followed by cropland > grassland > gully, while TN contents were in the order: forest > grassland > gully > cropland. Cropland soil showed significantly higher C:N ratio than other soils. It was not a surprise that forest soil had the highest C and N contents because of the accumulation of organic matter or plant tissue residues on the surface. The relatively high C:N ratio in cropland could reflect the influence of agriculture practices or result from soil N loss through soil erosion or plant uptake.

**Figure 3:**
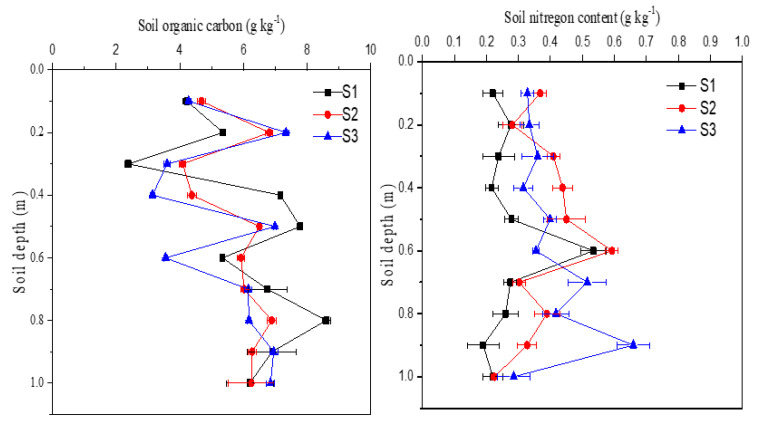
Depth-distributions of TOC and TN in sediment cores near the constructed dam (S1: upper-dam; S2: middle-dam; S3: bottom-dam positions).

Sediments had generally lower TOC and TN as compared with other soils, with a relatively higher C:N ratio (Table 2). The TOC range was 2.38 to 7.78 g kg^−1^ while TN 0.19 to 0.66 g kg^−1^ within the 1-m profile. There was no obvious content change with sampling depth and a little variation across the three dam positions (Figure 3). Similar C:N ratio in the sediments to cropland implied that cropland soil may be a primary C and N source in the sediments.

δ^13^C and δ^15^N compositions were found significantly different between soils and sediments (Table 2). Soil δ^13^C in forest was 1‰ to 5‰ lower than that of cropland and grassland soil, while soil δ^15^N in cropland was 1‰ to 2‰ higher than in grassland and forest. The range of δ^13^C or δ^15^N in source soils was −23.99 to −28.29‰ and 2.08 to 3.95‰, respectively.

**Figure 4:**
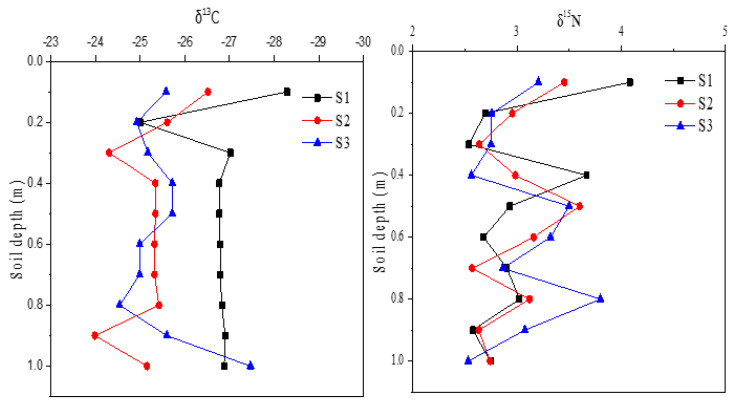
Depth-distributions of δ^13^C and δ^15^N in sediment cores near the constructed dam. (S1: upper-dam; S2: middle-dam; S3: bottom-dam positions).

Sediments contained the lowest δ^13^C and medium δ^15^N (Table 2). In overall, sediments at upper-dam position had lower δ^13^C than that at bottom-dam (Figure 4). δ^15^N measurement showed no apparent distribution pattern and were more variable than δ^13^C with sampling depth. But, in a few cores, δ^15^N was shown increasing with depth.

### 3.3. C-N Source Identification

**Figure 5:**
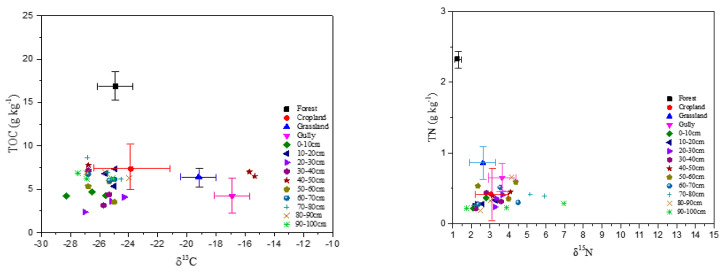
Correlations of TOC vs. δ^13^C and TN vs. δ^15^N in the source soils and sediments (Horizontal and vertical bars denote 95% confidence intervals).

**Figure 6:**
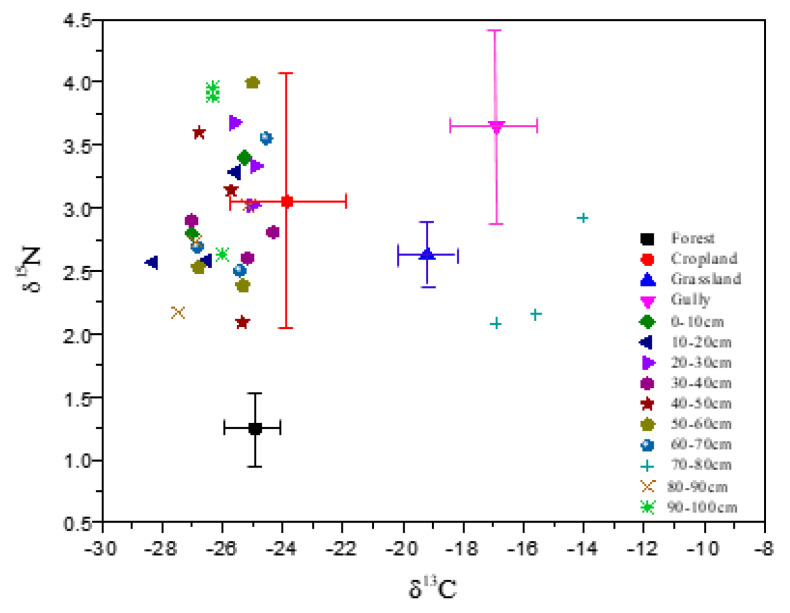
Correlation between δ^13^C and δ^15^N in the source soils and sediments. (Horizontal and vertical bars denote 95% confidence intervals).

Soil erosion or nutrient transport from upper landscapes to the depository zone near the constructed dam should have impacts on the C and N content of the sediments. Measurements of δ^13^C, δ^15^N, TOC and TN were found significantly different among source soils (Table 2). To explore the C and N source and contribution from source soils to the sediments, soil δ^13^C, δ^15^N, TOC, and TN measured under various land uses were tested and discriminated prior to analysis by the SIAR model. Correlations of δ^13^C vs. TOC and δ^15^N vs. TN in four source soils and sediments were presented in Figure 5. Data indicated that stable isotopes of δ^13^C and δ^15^N in cropland soil fell within TOC and TN clusters of the sediments, suggesting that C and N in sediments were more closely related to cropland soil. This finding was also confirmed by the correlation between δ^13^C and δ^15^N shown in Figure 6. Results demonstrated that cropland soil could be a primary contributor or source of C and N to the sediments; in other words, the C and N deposited in sediments were primarily from cropland soil in the watershed.

**Figure 7:**
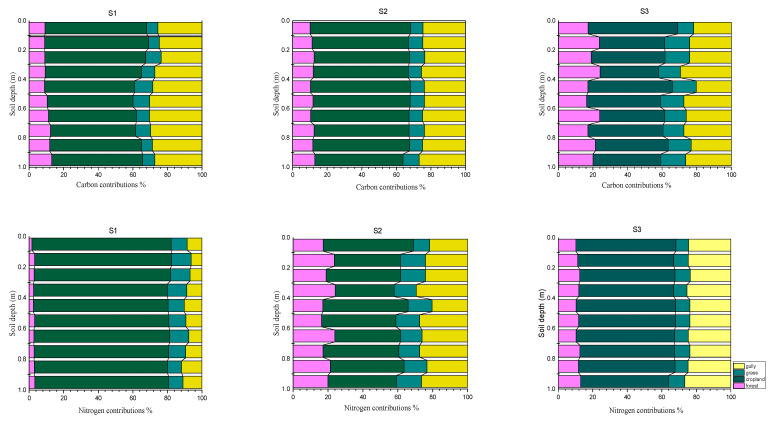
Percentage contributions of each source soils to C and N contents in sediment profiles deposited at three dam positions.

Cropland soil as a primary C and N source to the sediments was consistent with the estimates by the SIAR mixing model. In addition, the SIAR model also provided detailed estimates of the quantitative contributions of each source. The quantitative contributions of four source soils to C and N contents in sediment profiles at three dam positions were presented in Figure 7. Despite slightly, but non-significant, vertical changes of C and N contributions by each source across the sediment depth, data indicated that cropland contributed an average of 58.75% of sediment C, followed by gully (25.49%), grassland (6.49%), and forest (9.2%). Similarly, the contribution of sediment N was 80.58% by cropland, 10.30% by gully, 7.54% by grassland and 1.59% by forest. Thus, over 90% of sediment C and N were transported and deposited from cropland and gully or forest, and grassland contributed the least to C and N deposited in the sediments.

The construction of a dam at the outlet of the watershed was an important soil-water conservation practice that controlled soil erosion and retained soil nutrients. The variations of C and N contents in sediment profiles at three dam positions were consistent with water runoff pathways and could also reflect the history of soil erosion or C-N deposition and land use change in the watershed since the 1970s. The C and N contribution by forest cross-sediment profile was shown decreasing from the bottom-dam (S3) to upper-dam (S1) position, suggesting that the constructed dam retained more C and N from forest in runoff pathways as a result of water flow slowdown. In addition, increased forest area in the watershed as land use change by the conservation practices may result in less soil erosion or less C and N deposited near constructed dam. A higher contribution by cropland in the sediments at the upper-dam (S1) and middle-dam (S2) than bottom-dam position may reflect the influences of recent agriculture operations such as applications of fertilizers and manure or enhanced soil erosion or nutrient loss. Decreased C and N contribution by grassland toward the dam may result from increased grassland use or less soil nutrient loss. Gully was observed depositing more C and less N near the constructed dam. The contributions of each land use estimated by the SIRA model showed the potential of source contribution, but not the amounts of the contribution. Total C or N amounts of each source contribution to the sediments should be dependent upon soil erosion intensity, water flow rate and amounts, or erosion area, etc. Generally, higher C and N contents in source soils would result in a higher contribution to the sediments under the same erosion conditions.

### 3.4. C-N Correlation with Soil Properties

**Figure 8:**
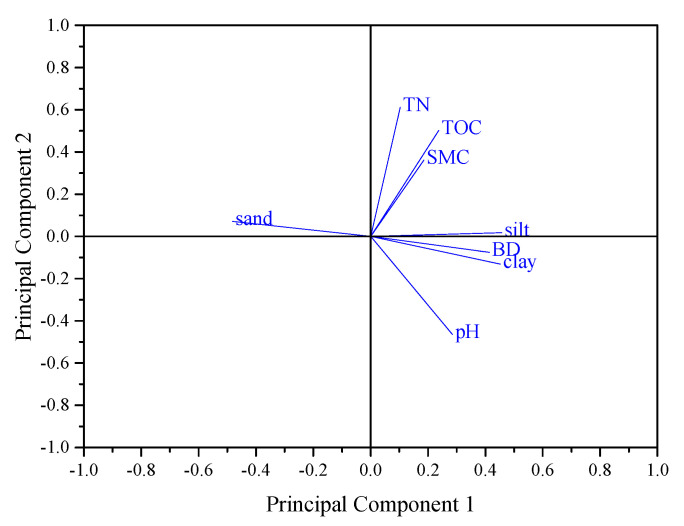
Principal component analysis showing two components associated with sediment C and N pools.

In order to further explore the relationship between C or N pools and other sediment properties, a principal component analysis was performed to identify the principle factors responsible for the C and N migration in the watershed. The analysis shown in Figure 8 demonstrated that two major components of sediment properties had accounted for 87.62% of total variance: the first component included TOC, TN, SWC, and silt content (58.73%), and the second component consisted of pH, BD, sand and clay content (28.89%). TOC and TN in sediments showed a significantly positive correlation with silt and clay content and a negative correlation with sand (*p* < 0.01), and the TOC and TN were also positively correlated with pH (*p* < 0.05). This suggested that the deposition conditions were also the major factors affecting soil C and N pool in the sedimentary zone, and soil C or N pool was more likely associated, migrated, and deposited with fine soil particles (silt and clay) during erosion processes.

## 4. Discussion

### 4.1. C-N Mobilization

It is well known that soil erosion results in large amounts of soil C and N transport to downstream [26]. Land uses in a watershed would certainly impact the C and N concentrations and deposition in sediment [27]. Data in Figure 7 indicated that cropland contributed averaged 58.75% and 80.58% of C and N in the sediments, respectively, and grassland contributed the least. TOC and TN contents in sediments of the watershed studied were shown generally lower than those of forest, cropland, and grassland soils (Table 2). Relatively low TOC and TN in the sediment retained by the constructed dam were comparable to those reported in similar small watersheds of the loess hilly-gully or Mediterranean region [15,28]. The discrepancy could be attributed to different migration processes of sediments at various spatial scales such as weather patterns, soil erosion type, landscapes, soils, or vegetation [6,29]. The type of soil erosion could affect the source of sediments and land use change and weather pattern would have significant impacts on the nature and erosion rate through their effects on hydrological and sedimentary dynamics within a watershed scale [30]. Therefore, TOC concentration and composition of the transported sediments may vary depending on the nature of major soil erosion processes [15,30,31].

Soil clay content was also identified as a major control factor for the depth-distribution of TOC and δ^13^C in soil profile [32]. Generally, higher TOC or OM in soil could be associated with higher clay content, because of surface adsorption, complexation, or aggregation by clay minerals. In addition, various erosion mechanisms in the hilly-gully watershed with complex terrestrial ecosystems may lead to C and N depletion in sediments relative to the source soils of the watershed.

This study site was located within the most severe soil erosion region. Few studies reported the characteristics of soil C or N loss in this area. But there were a few studies reported on the loess plateau area, which had similar soil characteristics to the watershed studied [33,34]. Considerable amounts of soil TOC were transported and redistributed through soil erosion every year in the hilly-gully region of western Liaoning, similarly to the loess plateau area, which had an important impact on the ecological sustainability and biogeochemical cycle in the region [35,36]. Data generated from this study verified that the most TOC loss primarily came from agricultural lands. Labile or active organic C in agricultural soils could be the fraction that was preferentially removed by erosion and deposited near the constructed dam [37]. This study demonstrated that dam construction at a watershed outlet would be an effective engineering approach to control soil erosion and retain considerable amounts of soil C or N transported by erosion processes [29,38,39].

### 4.2. C-N Source Control

This study showed that a stable isotope SIAR mixing model, as a reliable “fingerprint” tool, can be successfully employed to estimate the quantitative contribution of various C and N sources within a complex ecosystem, which could not be achieved by the mass balance mixed model [33]. In the studied watershed, topsoil of four potential source land uses (forests, cropland, grassland and gully) that were mobile and transportable by surface runoff were collected and analyzed by SIAR mixing model. Even though the watershed was dominantly under a gully and steep slope landscape, cropland was identified as a primary source of C and N loss to sediments, followed by gully, grassland, and forest (Figure 7). This finding was consistent with previous study by Liu et al. [40,41,42,43] and could be explained by agricultural management practices and government policies implemented in the region [44]. For instance, the family-based responsibility operation, which was established in 1984, is a basic management practice in rural China. Under this operation, certain area of cropland was allocated to each rural family for operation and management for at least 40 years, but cropland could not be transferrable on-market during the contract period. As family-based operations, farmer’s efforts are heavily focused on crop production and profitability with non-sustainable management practices, such as intensive land use operation, high chemical fertilizer application, and no return of plant residues to field after harvest. As a result, these management practices have resulted in less soil organic matter and poor soil quality, making soils more vulnerable to soil erosion. From 1978 to the present, soil erosion and soil C loss from sloping croplands was reported to increase due to rainfall and human activities, which highly contributed to soil sedimentation and nutrient deposition near constructed dam. In addition, soil erosion usually increased in gullies when a large amount of sediments moved with water flow [45].

Soil erosion usually has three stepwise processes: separation, transport, and deposition. The separation process first exposes organic matter-protected soil aggregates and clay minerals; subsequently, fine soil particles associated with organic C are preferentially moved from eroded lands and transported to and deposited at sedimentary sites [46]. In addition, human activities or operations had a significant impact on the source of sediment C and N and its deposition [18,47]. Vertical change of the C or N contributions by each source in the sediment profile could also reflect the temporal heterogeneity of soil erosion processes and land use changes [13,40,41]. In summary, conservation management practices, such as increasing forestry or grass lands, planting cover crops, adding soil organic matter, and less chemical fertilizer application, could help improve soil health and enhance soil resistance to erosion and nutrient loss under sloped landscapes.

### 4.3. Uncertainity Analysis

As shown in Figure 1, the sediment samples at S1 and S3 along the water flow pathway near the constructed dam of the watershed were collected. The estimates of the C and N deposition were based on samples collected near and sediments retained by the constructed dam. Due to the complex landscape features in the study area, it was difficult to accurately measure the length, width, and thickness of the sediment near the constructed dam, which may be the most important source of uncertainty for estimating sediment C or N concentration and retention [2,42]. In order to improve the assessment, more sediment profiles may be needed to better understand the geomorphological features of the sediments [42,43]. However, more sediment collection would be time-consuming, labor-intensive, and at high costs. In addition, when the stable C and N isotopes were used to identify the C and N sources in sediments, the potential sources of selected δ^13^C or δ^15^N component should be significantly different within the studied watershed, and the sediments within the range of δ^13^C or δ^15^ N isotope variation should also differ [44,45,46]. Furthermore, the impacts of soil erosion or post-deposition processes on the alteration of the isotope composition were not assessed [38,47,48], and the spatial variability of soil samples within landscape resulting from topography, agriculture activity, and frequent particle movement from ditches to dams was not considered, which could all potentially contribute to uncertainty of the estimation [40].

## 5. Conclusions

In this study, the SIAR mixing model that used soil ^13^C and ^15^N isotopes was successfully applied to quantitatively assess the extent of soil C or N loss from various land uses through soil erosion and identify the C or N sources to sediments deposited in a severely eroded watershed. Results indicated that TOC and TN of the sediments primarily came from cropland, followed by gullies, grasslands and forests. The C and N deposition in sediments near constructed dam at the watershed outlet varied vertically along the water flow pathway, which may be related to hydrodynamic conditions and land use change in the watershed. Significant correlations between TOC or TN content and soil properties in sediments showed that deposition conditions were the major factors affecting C and N pools in sedimentary zones. This study would provide a scientific insight of accurately assessing the C and N budget and developing watershed-specific best management strategies in a watershed scale.

## Figures and Tables

**Table 1:** Selected soil physiochemical properties at various land uses and landscape locations.

Land Use Type	Landscape Locations	Soil BD (g cm^−^^3^)	pH	Soil WC (g 100 g^−^^1^)	Soil Texture (g 100 g^−1^)		
					Clay	Silt	Sand
Forests	Upper	1.35 ± 0.21 a	7.51 ± 0.98 a	6.51 ± 0.21 ab	16.38 ± 1.23 a	34.23 ± 5.32 a	49.39 ± 8.63 bc
	Middle	1.36 ± 0.08 a	7.68 ± 1.23 a	6.70 ± 0.25 bc	15.23 ± 2.36 a	33.41 ± 4.02 a	51.36 ± 9.54 b
	Lower	1.38 ± 0.21 a	7.74 ± 1.03 ac	7.31 ± 0.17 b	19.87 ± 3.84 ab	35.78 ± 3.98 a	44.35 ± 4.53 b
Cropland	Upper	1.21 ± 0.11 bc	7.98 ±1.27 bc	5.23 ± 0.36 a	21.35 ± 1.68 b	41.28 ± 6.56 c	37.37 ± 1.69 a
	Middle	1.19 ± 0.12 c	7.86 ± 1.36 ab	5.21 ± 0.26 a	23.56 ± 3.65 bc	40.23 ± 4.32 bc	36.21 ± 2.67 a
	Lower	1.25 ± 0.09 bc	7.47 ± 0.96 a	4.62 ± 0.16 a	24.18 ± 4.65 bc	39.42 ± 5.63 b	36.4 ± 3.42
Grassland	Upper	1.2± 0.13 bc	8.1 ± 2.98 c	7.65 ± 0.42 bc	22.32 ± 2.36 b	41.36 ± 9.82 c	36.32 ± 5.49 a
	Middle	1.2 ± 0.18 bc	7.9 ± 2.10 bc	7.58 ± 0.26 bc	26.31 ± 2.42 c	41.32 ± 4.45 c	32.37 ± 6.31 a
	Lower	1.27 ± 0.05 bc	8.2 ± 1.63 c	8.2 ± 0.35 c	26.35 ± 5.61 c	39.23 ± 6.23 bc	34.42 ± 5.22 a
Gully	Plain	1.18 ± 0.15 c	8.03 ± 0.65 c	12.21 ± 0.65 d	26.31 ± 4.36 c	38.56 ± 6.26 b	35.13 ± 3.43 a

Note: all values represented mean ± standard deviation. Different letters indicated significant differences among land uses and landscape positions at the *p* < 0.05 level.

**Table 2:** Measurement of total organic carbon (TOC), total nitrogen (TN), C:N ratio and stable isotope composition in various source soils and sediments.

Types	TOC (g kg^−1^)	TN (g kg^−1^)	δ^13^C (‰)	δ^15^N (‰)	C/N Ratio
Forest	16.86 ± 0.06 a	2.33 ± 0.06 a	−24.9 ± 0.25 a	1.25 ± 0.06 d	7.24 c
Cropland	7.35 ± 0.04 b	0.52 ± 0.04 c	−23.85 ± 0.43 b	3.05 ± 0.05 ab	14.14 a
Grassland	6.43 ± 0.09 b	0.86 ± 0.09 b	−19.2 ± 0.25 bc	2.63 ± 0.03 c	7.48 b
Gully	4.21 ± 0.07 c	0.65 ± 0.02 c	−24.9 ± 0.4 a	3.65 ± 0.02 a	6.48 c
Sediments	5.79 ± 0.05 bc	0.40 ± 0.09 d	−25.2 ± 0.3 a	2.92 ± 0.02 c	14.48 a

Note: all values represented mean ± standard deviation. Different letters indicated significant differences among source soils and sediments at the *p <* 0.05 level.

## Data Availability

Data will be available upon request.
